# Cortical Contribution to Linear, Non-linear and Frequency Components of Motor Variability Control during Standing

**DOI:** 10.3389/fnhum.2017.00548

**Published:** 2017-11-10

**Authors:** Niklas König Ignasiak, Lars Habermacher, William R. Taylor, Navrag B. Singh

**Affiliations:** Department of Health Sciences and Technology, Institute for Biomechanics, ETH Zürich, Zürich, Switzerland

**Keywords:** Hoffman-reflex, postural sway, sample entropy, DFA, lyapunov exponent, motor output variability, cognition, dual task

## Abstract

Motor variability is an inherent feature of all human movements and reflects the quality of functional task performance. Depending on the requirements of the motor task, the human sensory-motor system is thought to be able to flexibly govern the appropriate level of variability. However, it remains unclear which neurophysiological structures are responsible for the control of motor variability. In this study, we tested the contribution of cortical cognitive resources on the control of motor variability (in this case postural sway) using a dual-task paradigm and furthermore observed potential changes in control strategy by evaluating Ia-afferent integration (H-reflex). Twenty healthy subjects were instructed to stand relaxed on a force plate with eyes open and closed, as well as while trying to minimize sway magnitude and performing a “subtracting-sevens” cognitive task. In total 25 linear and non-linear parameters were used to evaluate postural sway, which were combined using a Principal Components procedure. Neurophysiological response of Ia-afferent reflex loop was quantified using the Hoffman reflex. In order to assess the contribution of the H-reflex on the sway outcome in the different standing conditions multiple mixed-model ANCOVAs were performed. The results suggest that subjects were unable to further minimize their sway, despite actively focusing to do so. The dual-task had a destabilizing effect on PS, which could partly (by 4%) be counter-balanced by increasing reliance on Ia-afferent information. The effect of the dual-task was larger than the protective mechanism of increasing Ia-afferent information. We, therefore, conclude that cortical structures, as compared to peripheral reflex loops, play a dominant role in the control of motor variability.

## Introduction

Motor variability is an inherent feature of all human movements and serves as an indicator for the quality of functional movement performance such as walking or standing (Konig et al., 2016). During aging, as well as in pathological cohorts, the ability to perform these fundamental functional movements is diminished, therefore confining safe mobility and consequently limiting the maintenance of an independent and self-determined life style 2001. A better understanding of the etiology as well as the control of motor variability during functional tasks is therefore imperative in order to develop adequate treatment regimens and to allow sensitive identification of pathological motor performance.

Standing balance is generally assessed by quantifying postural sway (PS)—an expression of motor variability—because of the clear relationship between the position of the body center of mass (CoM) and the base of support (BoS), which defines the biomechanical stability of a system (Winter, 2009). It is therefore plausible that the primary aim of the human sensory-motor system (HSMS) is to minimize PS in order to maintain the CoM within the boundaries of the BoS, currently determined using margins of stability approach (Hof et al., 2005). In this context, PS has been seen as an unwanted side-product of movement production and was thus understood to be the result of noisy signaling processes within the HSMS, including e.g., sensory perception at the receptor, information transmission via neural signaling, and motor-neuron firing (Faisal et al., 2008). Consequently, the clinical interpretation of PS has been that elevated levels of postural variability are indicative of poor motor performance and pathological functioning of the HSMS. This general concept of sway-minimization has recently been refined by the notion that the HSMS might not globally aim to reduce all dimensions of motor variability, but rather only those that interfere with the success of the movement goal (Scholz and Schoner, 1999; Todorov and Jordan, 2002). Moreover, the HSMS might only minimize motor variability to an extent that is required to fulfill the motor task, rather than to an absolute minimum, in order to avoid excessive control efforts (Todorov and Jordan, 2002).

More recent evidence suggests that motor variability is not necessarily the result of “erroneous” motor control processes, but instead is an important prerequisite for successful task performance. It has been postulated that an optimal level of variability is required that balances motor behavior between being excessively variable (and thus unstable) and being too rigid, and thus not sufficiently flexible for adequate motor performance (Stergiou et al., 2006). To support this claim, it has been shown that motor variability changes over the course of motor learning in a U-shaped manner (Wilson et al., 2008; Fetters, 2010; Kyvelidou et al., 2013), can be regulated depending on the requirements of the motor task at hand (Loram et al., 2001; Mitra and Fraizer, 2004; Wu et al., 2014; Pekny et al., 2015), and might facilitate the gathering of sensory information (Carpenter et al., 2010). This notion of flexible regulation of motor variability, however, indicates that the HSMS is at least partially in control of the magnitude of motor variability that occurs during a specific motor task.

In a recent publication, we investigated the influence of peripheral Ia-afferent inputs from the Soleus muscle (SO) for the regulation of motor variability during upright standing (König et al., 2017). Beside a small contribution of the Ia-afferent pathway, we found indirect evidence for the involvement of supraspinal structures during the regulation of motor variability. In addition, dual-task experiments provide further evidence for the involvement of cognitive resources for the control of PS (Woollacott and Shumway-Cook, 2002). Here, two competing concepts of *resource-competition* and *adaptive resource-sharing* provide a possible explanation for the opposing findings of increased (Mitra, 2003; Pellecchia, 2003) or decreased (Hunter and Hoffman, 2001; Andersson et al., 2002) PS under dual task conditions (Mitra and Fraizer, 2004). In either case, there is convincing evidence for the direct involvement of the cerebral cortex, via corticospinal pathways, for the control of standing balance (Jacobs and Horak, 2007). Whether postural control is primarily governed by automatized peripheral spinal control loops or by supraspinal resources seem to depend on the demand of the postural task. It is hypothesized that in more challenging posture conditions, increased drive of supraspinal centers acts via pre-synaptic inhibition to supress automatized peripheral spinal loops (Taube et al., 2008a), and thereby shift the control process from a peripheral to a cortical driven mechanism. Consequently, the investigation of cortical and peripheral control processes during different PS tasks, might help to unravel how the HSMS is able to flexibly control motor variability.

Based on the concept of optimal variability, the goal of this study was therefore to extend the previous findings on the contribution of peripheral Ia-afferents and to comparatively assess the role of cognitive resources in the control of motor variability. We hypothesized, that (i) subjects will be able to significantly reduce motor variability by increasing voluntary attention on the postural task, (ii) that this attention effect will be at least partially reduced by an additional cognitive task (thereby highlighting a role of the cerebral cortex in variability control), and (iii) that voluntary reduction of PS can be realized by pre-synaptic inhibition of the peripheral Ia-afferent loop. In order to address these questions, we aimed to investigate whether focusing attention on the control of PS allows sway to be voluntarily minimized, and thereby establish to what extent motor variability can be flexibly regulated by the HSMS. In complementing these measurements with a dual-task paradigm, the role of cognitive resources for this control process was assessed, hence also allowing an understanding of whether this cortical control strategy becomes effective by inhibiting automatized peripheral control mechanisms.

## Methods

Twenty physically and mentally healthy volunteers (10 male/10 female; with mean ± standard deviation (*SD*): 22.1 ± 2.5 years; 174.6 ± 9.3 cm; 68.9 ± 12.5 kg) were recruited from the local community to perform multiple standing trials while their PS and H-reflex (HR) were evaluated. The study was approved by the institutional ethics committee and was performed in accordance with the Declaration of Helsinki. All subjects provided written informed consent prior to participation in the study.

Each participant performed five different standing conditions; standing naturally with eyes open (SNEO), standing naturally with eyes closed (SNEC), minimizing sway with eyes open (MSEO), minimizing sway with eyes closed (MSEC), and finally minimizing sway while subtracting sevens (i.e., dual task) with eyes open (DTEO). In each condition, PS was recorded during three repetitions of 1 min standing trials. In each condition, an additional standing trial was performed to assess the HR. Between trials subjects were requested to sit down on a chair in order to avoid fatigue. All conditions and trials were presented in a randomized order. During trials, participants stood barefoot on a force plate (Kistler, Winterthur, Switzerland; sampling frequency 1,000 Hz), with their feet together and the arms crossed in front of the thorax. During trials with SNEO conditions, participants were requested to focus on a picture presented on the monitor placed at eye height and ~2 m in front of the participants. During trials with minimization conditions with eyes open, namely MSEO, and DTEO conditions, participants were requested to stand as in the case of SNEO with additional instructions to try and “minimize sway as much as possible.” In both MSEO and DTEO conditions, additional motivation was provided by a reward offering them 5 CHF (Swiss Francs) for every trial when participants were able to successfully maintain sway within an area of 10 × 10 mm^2^ for the entire duration of the trial. Finally, in the sway minimization with eyes closed condition, MSEC, participants were requested to stand quietly with eyes closed and try and “minimize sway as much as possible”, while being offered a monitory reward of 5 CHF for every trial where they were able to maintain sway within an area of 15 × 15 mm^2^.

At the beginning of the measurement session, subjects performed a series of 1 min familiarization trials while standing relaxed. These trials also served to identify mean AP sway for each participant. The location of the feet relative to the force plate was kept constant throughout the testing session. Afterwards, subjects received real-time visual feedback of their own PS (i.e., location of the center of pressure, CoP, in anterior/posterior, AP, and medio/lateral, ML, directions was displayed on a dartboard-like representation with the center being the mean AP and ML locations in the vertical and horizontal axes respectively) in order to make subjects aware of their own PS and to allow them to develop strategies for sway minimization. In addition to the familiarization session at the beginning of the measurement session, each subject also performed at least three training trials for only the MSEO condition, or as many as they required to feel confident to be able to actively reduce sway. Once the participants felt confident about their ability to actively reduce sway no further training was provided for the other minimization conditions, DTEO and MSEC. Finally, a full HR recruitment curve was recorded during standing in order to identify stimulation intensities at which maximum H and M amplitudes occurred. Later, during the testing trials, HRs were elicited at the ascending edge of the HR recruitment curve.

### H-reflex measurements

Before placing the EMG or stimulation electrodes, relevant skin areas were shaved, abraded with preparation gel (Nuprep, NR Sign Inc., Canada), and cleaned with water to ensure a low skin impedance (Robinovitch et al., 2002). The wireless EMG electrodes (Trigno, Delsys, United States) were placed on the Soleus (SO) and the Tibialis anterior (TA) muscles according to the SENIAM protocol (Hermens, 1999). The HR stimulation electrode was attached while the subjects assumed a prone position. The cathode (8 mm diameter, Ag/AgCl) was moved within the popliteal fossa of the right leg until the largest H-response without an M-response could be evoked (Hermens, 1999). Once located, this area was marked, and the electrode was fixed with tape and a non-elastic Velcro strap to prevent movement during the measurement. The anode (40 × 90 mm, Spes Medica, Italy) was placed 2 cm proximally of the patella on the body.

As HR response depends on the body position during PS (Palmieri et al., 2004), the mean sway position in the anterior-posterior (AP) direction was set as the sway-threshold (Nexus, VICON, United Kingdom) in order to trigger the HR-stimulation. Subjects were stimulated using a constant-current stimulator (DS7A, Digitimer, United Kingdom), which was only triggered when the participant swayed in an anterior direction crossing the sway-threshold value, thereby ensuring similar muscle geometry in each stimulation condition. Additionally, a minimal inter-stimulus interval of 8 s was used to avoid post-activation depression (Tokuno et al., 2008). To obtain full a H/M-recruitment curve, stimulus intensity (0.5 ms square-wave-pulses) was increased in increments of 1 mA until a full recruitment curve was retrieved.

The sampling frequency of the EMG was set at 2 kHz and the signal was band-pass filtered (10–500 Hz, Butterworth 2nd order). The recording window to obtain the background EMG (bEMG), was set at 50 ms prior to each stimulus (Chen and Zhou, 2011). The bEMG recordings as well as the H and M responses were later assessed offline using a custom code (Matlab, Mathworks, Natick, MA, USA). In order to consistently evaluate HR amplitudes at the ascending edge of the HR recruitment curve across different standing conditions, the intensity of stimulation was kept constant at 80–90% of the intensity at H-max (Crone et al., 1990). For each condition, five stimulations at this intensity were administered. After confirming absence of muscular pre-activation (bEMG: RMS < 0.05 mV) the mean of all HR amplitudes (HRX) was calculated. Furthermore, in order to ensure a constant test afferent volley, three stimulations at the intensity of M-max were randomly administered during the HR recording trials.

### Postural sway measurements

PS, was measured during 1 min standing trials on the force plate with no stimulation applied, according to the protocols described above. The initial and final 5 s of each trial of the CoP data were removed in order to avoid transients. Before calculating linear and frequency parameters, all data were band-pass filtered (0.75–35 Hz, Butterworth 4th order) and detrended. PS parameters were calculated for the entire sway signal in the resultant (R) and the AP directions separately, to ensure quantification of postural sway along the dimensions of the soleus muscle's main function. In order to quantify the magnitude of PS, multiple linear parameters were quantified, including sway area, velocity, and distance of CoP travel. The frequency content of the signal was evaluated by assessing mean and median power frequency, as well as the absolute power within three frequency bands (low: < 3 Hz; medium: 3–10 Hz; high: 10–30 Hz). In addition, the temporal structure of sway was assessed using three non-linear parameters. Here, the raw-data (no filter applied) was down-sampled to 100 Hz before the following parameters were calculated: alpha after detrended fluctuation analysis (DFA) (Konig et al., 2014), sample (SE) and approximate entropy (AE) (Duarte and Sternad, 2008), and largest Lyapunov exponent (LyE) (Yentes et al., 2013). For the measures of SE and AE, the input parameters (vector length m = 2; tolerance r = 0.2 × *SD*) were kept constant across all trials, after confirming that results were insensitive to other m/r combinations. To determine LyE, the Wolf algorithm was applied (Ladislao and Fioretti, 2007) with number of embedded dimensions (Dim) and time lag (tau), which were calculated as the sample median of all trials (van Schooten et al., 2013), and kept constant (Dim = 5 and tau = 5) across all trials in order to be consistent with our previous study (König et al., 2017).

### Factor analysis

In total, 25 PS parameters were calculated. In order to reduce the dimensionality of the dataset, factor analysis (FA) using the “VARIMAX” procedure was applied, where each condition for all subjects (*n* = 20) was considered to be a case (totalling 100 cases). The Kaiser-Meyer-Olkin (KMO) criterion was used to extract the appropriate number of components with Eigenvalues >1 (Tabachnick and Fidell, 2013). To ensure consistency of the original measure, the following criteria were applied in order to remove individual parameters from the analysis: (a) measures of sampling adequacy <0.5, (b) measures with communality <0.5, and (c) measures that caused complex structure (i.e., correlations >0.4 in two or more components) (Tabachnick and Fidell, 2013). Z-scores of components derived from FA were used for further analysis as well as for interpretation.

### Inferential statistics

Three mixed-factor repeated measure ANCOVAs were conducted separately to explore the relationship between each of the three dependent sway components and the independent condition variable (five levels: SNEO, SNEC, MSEO, MSEC, DTEO). In order to investigate the contribution of the HR on the dependent variables, HRX was included as a covariate in the model. Furthermore, in order to ensure consistency of the test afferent volley, a mixed-model repeated measure ANOVA was conducted on the recorded M-max values, with conditions as fixed and subjects as random factors. The alpha level for all tests was set at 5% and *post-hoc* comparisons were conducted using Sidak adjustment for multiple comparisons. Values greater than three standardized scores (*Z*-scores) were considered to be outliers, and were removed from the analysis. All statistical analyses were performed in SPSS (SPSS 23, IBM, Armonk, NY, USA).

## Results

### Factor analysis

After seven iterations, the final solution for the FA approach was achieved, revealing three components that loaded eight of the initial parameters (Table 1). These three components explained 93% of the total variance in the initial dataset (KMO = 0.684, Bartlett-Test of sphericity < 0.001). The first component included the parameters SE, AE, and LyE and was interpreted as a non-linear sway component (NSC). The second component comprised the measures of sway distance and velocity, and was interpreted as a linear sway component (LSC). The third component contained both parameters of mean frequency for the entire sway data the anterior-posterior direction and was therefore interpreted as a frequency sway component (FSC).

**Table 1:** Summary of the retrieved FA components, displaying the communalities, explained variance by the components, and the loading of the different measures on the component.

**Parameter**	**NSC**	**LSC**	**FSC**	**Communalities**
meanDist-R		0.927		0.936
meanDist-AP		0.939		0.960
meanFreq-R			0.952	0.934
meanFreq-AP			0.955	0.956
SE-AP	0.964			0.963
AE-AP	0.966			0.967
LyE-AP	0.927			0.868
peakVel-AP		0.903		0.898
Total variance explained (%)	41.89	28.90	22.59	

*Only loadings >0.4 are displayed. Dist, distance; Vel, Velocity; Freq, frequency; R, resultant; AP, Antero-posterior direction*.

### Inferential statistics

Comparison of the M-max amplitudes across conditions was not significant [*F*_(4, 25)_ = 1.07, *p* = 0.4], indicating a consistent test afferent volley and therefore stable recruitment of Ia-afferents.

**Figure 1:**
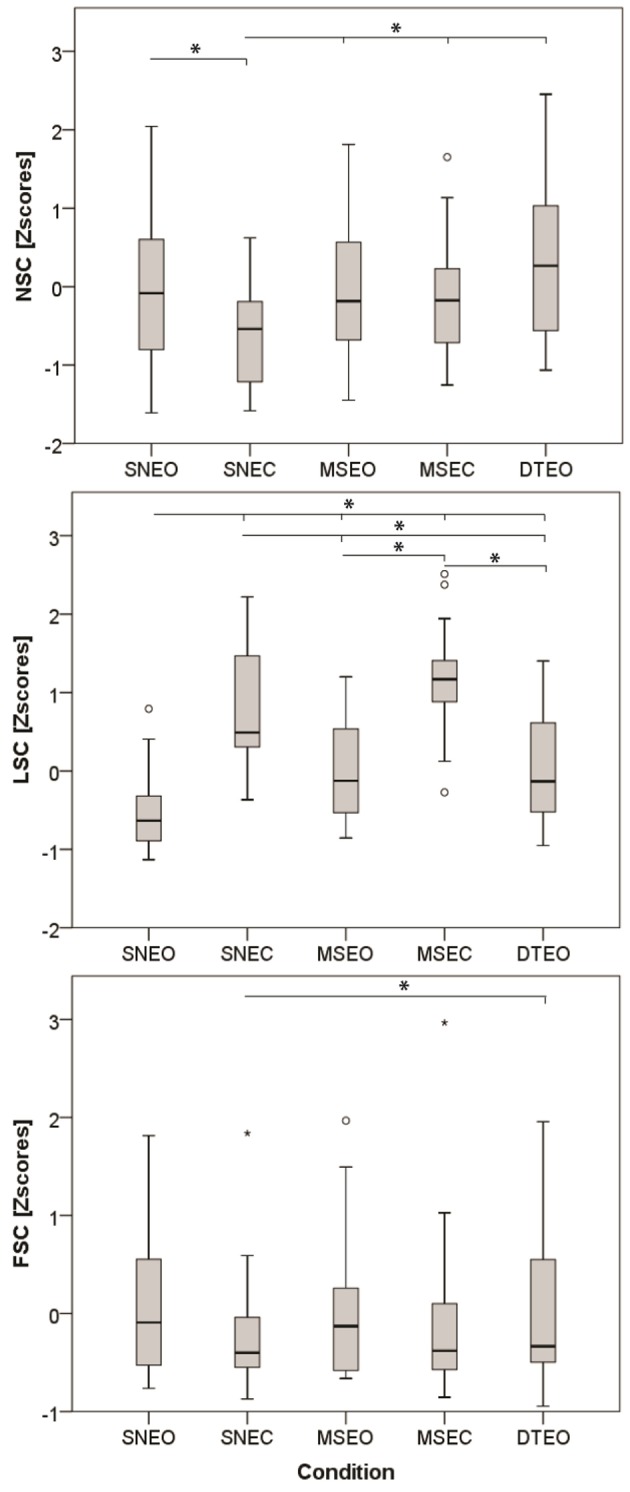
Effect of the condition (SNEO, normal standing eyes open; SNEC, normal standing eyes closed; MSEO, minimized sway eyes open; MSEC, minimized sway eyes closed; DTEO, dual task eyes open) on the three dependent variables. Asterisk indicates significant effects at *p* < 0.05.

The independent condition variable had a significant effect on all three dependent variables: NSC [*F*_(4, 23)_ = 10.7, *p* < 0.001], LSC [*F*_(4, 23)_ = 39.2, *p* < 0.001] and FSC [*F*_(4, 21)_ = 10.7, *p* < 0.001]. The *post-hoc* comparison revealed that NSC in the SNEO was larger than the SNEC condition (Δ = 0.62 [0.18–1.06]^1^; S.E. = 0.14; df = 19.28; *p* = 0.003) (Figure 1). NSC in the SNEC condition was lower than in all other conditions: MSEO (Δ = 0.63 [0.19–1.06]; S.E. = 0.14; df = 23.04; *p* = 0.002), MSEC (Δ = 0.44 [0.28–0.85]; S.E. = 0.13; df = 17.75; *p* = 0.03), and DTEO (Δ = 0.92 [0.42–1.41]; S.E. = 0.16; df = 23.33; *p* < 0.001).

*Post-hoc* comparisons for the second component revealed that LSC values were lowest in the SNEO compared to all other conditions: SNEC (Δ = 1.40 [0.92–1.88]; S.E. = 0.16; df = 26.25; *p* < 0.001), MSEO (Δ = 0.55 [0.14–0.97]; S.E. = 0.13; df = 23.34; *p* = 0.004), MSEC (Δ = 1.78 [1.27–2.28]; S.E. = 0.17; df = 28.55; *p* < 0.001), and DTEO (Δ = 0.63 [0.07–1.19]; S.E. = 0.18; df = 20.34; *p* = 0.02). Furthermore, conditions with eyes closed had significant larger LSC values compared to the dual-task condition (SNEC vs. DTEO: Δ = 0.77 [0.22–1.33]; S.E. = 0.18; df = 29.55; *p* = 0.002|MSEC vs. DTEO: Δ = 1.15 [0.58–1.72]; S.E. = 0.19; df = 30.21; *p* < 0.001).

**Figure 2:**
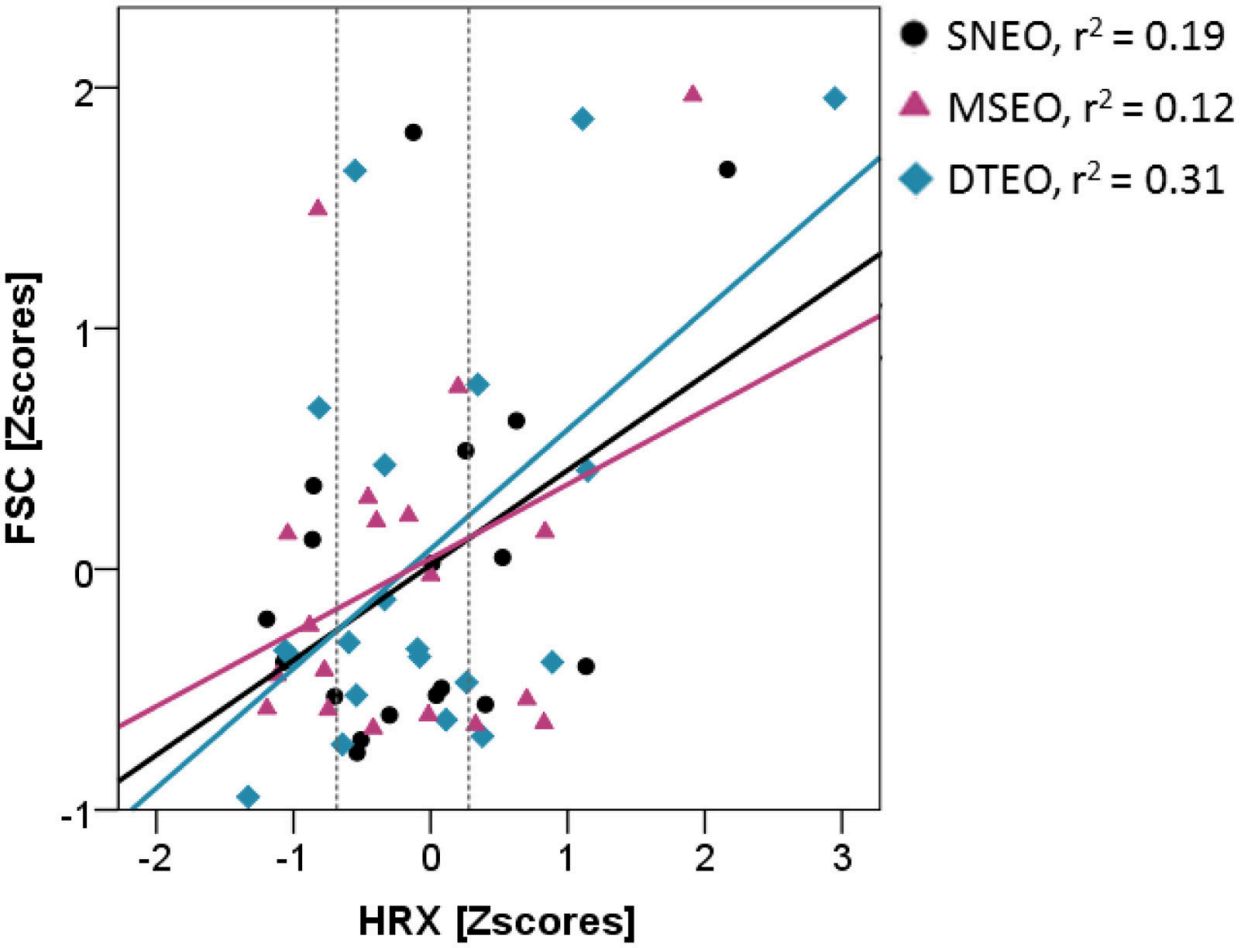
Association between HRX and FSC for the three conditions with eyes open. Vertical dashed lines indicate 25 and 75th percentiles of HRX.

The FSC was significantly elevated in the DTEO compared to the SNEC (Δ = 0.23 [0.02–0.55]; S.E. = 0.08; df = 16.02; *p* = 0.03). Furthermore, there was a significant interaction effect of condition^*^HRX (Table 2, Figure 2). During the DTEO condition the correlation between FSC and HRX was increased (*r*^2^ = 0.31; *p* = 0.01) compared to the SNEO (*r*^2^ = 0.19; *p* = 0.07) and MSEO (*r*^2^ = 0.12; *p* = 0.16) conditions. The interactive effect size of condition and HRX on FSC was 0.04.

**Table 2:** Results of the ANCOVA for repeated measures with the sway components as dependent variables, HRX as the independent variable, and condition as a fixed factor.

		**NSC**					**LSC**					**FSC**				
	**N-Df**	**SS**	**D-Df**	**F**	**SIG**	**$\eta_{\text{G}}^{2}$**	**SS**	**D-Df**	**F**	**SIG**	**$\eta_{\text{G}}^{2}$**	**SS**	**D-Df**	**F**	**SIG**	**$\eta_{\text{G}}^{2}$**
Condition	4	**9.31**	**20.64**	**12.47**	**<0.01**	**0.11**	**36.32**	**22.86**	**36.81**	**<0.01**	**0.42**	**2.97**	**19.23**	**10.99**	**<0.01**	**0.04**
HRX	1	0.01	67.02	0.01	0.63	0.00	0.00	47.64	0.02	0.89	0.00	0.01	67.63	0.46	0.50	0.00
Condition ^*^ HRX	4	1.83	23.48	1.89	0.45	0.02	1.30	24.74	1.36	0.28	0.03	**2.32**	**20.89**	**9.96**	**<0.01**	**0.04**
Error	16.33						18.59					6.60				
C. Total	85.19						88.24					56.69				

*SS, Sum of Squares; N-df, Numerator degrees of freedom; D-df, Denominator degrees of freedom; and $\eta_{\text{G}}^{2}$ = Generalized eta-squared. Bold indicates significant effects at p < 0.05*.

## Discussion

The aim of this research was to investigate the contribution of peripheral Ia-afferents and assess the role of cognitive resources in controlling motor variability. We hypothesized that active attention on PS would allow subjects to reduce motor variability; that this effect is reduced by an additional cognitive dual task; and finally, that tightened control of sway is achieved by supressing other peripheral control loops (i.e., Ia-afferents). In this study, however, subjects were not able to voluntarily reduce motor output variability, as can be seen by an increase in the linear sway component, LSC, in the MSEO compared to the SNEO trials, despite financial rewards ensuring high motivation. In other studies with similar cohorts, subjects were able to voluntarily reduce PS (Loram et al., 2001; Richer et al., 2017a). However, in these studies subjects were instructed to focus on the movement around the ankle joint rather than the movement of the CoP, which might account for the inconsistent findings. It appears that the CoP movements are not simply the representations of the CoM but might also fulfill other sensory acquisition functions (Murnaghan et al., 2011; Takeda et al., 2017). Our results indicate that the healthy cohort performed standing with near-minimum variability, with the remaining variability probably resulting from the neuromuscular noise (Singh et al., 2012), and therefore do not support the hypothesis of flexible motor variability control by the HSMS.

In a similar manner to LSC, there was little or no effect on the NSC between *minimize sway* (MS) and the SNEO trials. The NSC was comprised of the positively correlated measures SE, AE, and LyE and were therefore interpreted in a manner indicating that lower NSC values represent an increase in regularity (i.e., entropy measures) (Rigoldi et al., 2013; Yentes et al., 2013; van Emmerik et al., 2016) and stability (i.e., lyapunov exponent) (Stergiou et al., 2006; Ladislao and Fioretti, 2007; van Emmerik et al., 2016) of the PS (König et al., 2017). It was expected that with increased attention during the MS conditions, PS would become more regular (i.e., lower NSC values), due to the known association between attention and sway regularity (Donker et al., 2007; Rigoldi et al., 2013; Schniepp et al., 2013). However, this was not the case in our study. Interestingly, a subsequent re-analysis of data with sway parameters only in AP directions revealed that LSC and NSC remained unchanged in the MS conditions. Taken together, the results of the inability to reduce LSC and the unchanged NSC in the MS trials might indicate that our subjects did not attempt to reduce PS by increasing attention on the task, but rather applied other unsuccessful strategies (on average only 2.6 out of 12 possible MS trials were performed successfully by each subject). One potential strategy might have been to increase muscular co-contraction and thereby to attempt to stiffen their posture (Carpenter et al., 2001; Loram et al., 2001). As can be seen in the LSC values, eye closure has a strong effect on PS (Prieto et al., 1996; Taube et al., 2008b). The reduction of NSC during SNEC trials might indicate a “naturally” occurring enhanced attention on PS when eyes are closed (Donker et al., 2007; Rigoldi et al., 2013; Schniepp et al., 2013). However, when comparing MSEO and MSEC trials to SNEO a reduction in NSC was not observed. A reduction in NSC would have implied increased attention via central resources (Donker et al., 2007; Rigoldi et al., 2013; Schniepp et al., 2013; van Emmerik et al., 2016) and therefore no change in this parameter provides further support for the hypothesis that a peripherally originated co-contraction type control was applied when attempting to reduce sway. It is common practice to conduct quiet standing tasks under minimize sway conditions (Prieto et al., 1996). However, as our participants could not effectively harness the attentional resources during MS conditions, and the fact that the knowledge of the motion of CoM is critical in actively modifying the movements of CoP, care must be taken in considering the use of minimize sway conditions.

### Effect of dual-task

In line with previous research, the dual-task had a significant effect on PS, as could be observed in the elevated levels of LSC in the DTEO compared to the SNEO condition (Mitra, 2003; Pellecchia, 2003). Furthermore, there was a trend of the NSC to become less regular and more unstable in the dual-task condition (i.e., larger NSC values). This is in line with the hypothesis that entropy measures quantify the involvement of attention for the postural task and as the attentional resources were used for the subtracting task and could not be allocated for the control of posture (i.e., resource-competition) (Woollacott and Shumway-Cook, 2002; Mitra and Fraizer, 2004; Donker et al., 2007; Rigoldi et al., 2013; Schniepp et al., 2013). However, contrary to the results presented here, it has also been reported that cognitive load might decrease linear sway magnitude (Hunter and Hoffman, 2001; Andersson et al., 2002). It has been argued that this might be achieved by a shift toward a more “automatized” control of sway, together with a higher frequency sway pattern (Wulf et al., 2001; Richer et al., 2017b). Furthermore, it has been shown that this process might be more effective in improving postural stability compared to the active attention-based reduction in sway (Richer et al., 2017b). In either case, no consensus has been reached whether additional cognitive dual-task loads during postural tasks are purely destabilizing due to resource-competition or alternatively, facilitates the performance of the cognitive task (i.e., adaptive resource-sharing) (Mitra and Fraizer, 2004). In this interpretation, the elevated level of LSC in the dual-task condition would not be understood as the lack of control resources, but rather the result of “deliberate” sway increase in order to facilitate the cognitive task. However, it has been argued that a “deliberate” increase in PS might only occur during supra-postural tasks, such as those that require precise perceptual information acquisition (Stoffregen et al., 2007). Furthermore, some of the study participants reportedly found it difficult to focus on the dual cognitive load while simultaneously managing to maintain posture. Therefore, it would appear that perception is unlikely to facilitate the cognitive subtraction task, rather the increase in LSC during DTEO could be interpreted as a destabilizing effect due to resource-competition.

### Role of ia-afferent information

The integration of Ia-afferent information for the control of balance was quantified by the HRX. It was hypothesized that an elevated drive of cognitive resources by voluntary attention on the postural task would suppress the integration of simple peripheral reflex loops. In the current study, no posture stabilizing effect through additional cognitive resources was achieved (compare SN to MS trials) and thus no effect of facilitation or inhibition on the reflex pathway occurred. However, the ANCOVA revealed a significant interaction effect between condition and HRX for the FSC. The change of regression slope between FSC and HRX is therefore indicative of a change in control strategy, since the relationship between the two variables changes. Here, it appears that in the DTEO condition, the association between FSC and HRX is larger than in other conditions. The positive correlation indicates that a larger HRX is associated with a higher mean sway frequency. Sway frequency, in return, is inversely associated with sway magnitude (c.f. LSC and FSC for EO and EC trials) (Carpenter et al., 2001; Wulf et al., 2001; Richer et al., 2017b). Therefore, HRX serves in a mediating manner to minimize the destabilizing effects of the cognitive dual-task on PS (i.e., increase in LSC). The generalized effect size of HRX on FSC was small, with an eta-square value of 0.04. This contribution of Ia-afferent information on postural performance is, however, strikingly similar to the results of a previous study (also 4%) in which increased reliance on Ia-afferents balanced out the destabilizing effects of eye closure (König et al., 2017). Therefore, it appears that increased reliance on Ia-afferents can in general counter-balance postural destabilizing effects by~ 4%. While a 4% contribution might appear small, its relevance lies in the fact that this contribution is occurring from one muscle. While maintaining balance during MS conditions, participants likely resorted to a co-activation control strategy. It is likely that evaluating co-activations from multiple muscles in both legs might provide a better understanding of how posture is maintained (Danna-Dos-Santos et al., 2007, 2015; Saffer et al., 2008; Boonstra et al., 2015) and finally, how co-activation of multiple muscles influences the contribution of overall Ia-loops toward postural control.

Although the study had a moderate sample size and some participants reportedly found dual-task paradigm challenging to manage, our results clearly showed that voluntary focus on minimizing PS did not lead to a further reduction of the magnitude or temporal structure of motor variability, suggesting that the healthy young subjects in this study performed standing with minimum or near-minimum motor variability in the SNEO condition. However, during the dual-task paradigm, a destabilizing effect on the balance was observed, which indirectly indicates a contribution of cognitive resources on the control of motor variability. Here, enhanced reliance on Ia-afferent information can reduce these destabilizing effects by up to 4%, which has been supported previously by similar findings on the contribution of this reflex loop on motor variability control. Comparatively, the destabilizing effect of the dual-task was larger than the protective effect of the Ia-afferent loop, which ultimately indicates a dominant role of cortical compared to peripheral neurophysiological resources for motor variability control.

## Author contributions

All authors listed have made a substantial, direct and intellectual contribution to the work, and approved it for publication.

### Conflict of interest statement

The authors declare that the research was conducted in the absence of any commercial or financial relationships that could be construed as a potential conflict of interest.

## Footnotes

^1^Values in square brackets are 95% confidence intervals for the value of Δ.
